# Underestimated Properties of Nanosized Amorphous Titanium Dioxide

**DOI:** 10.3390/ijms23052460

**Published:** 2022-02-23

**Authors:** Marek Wiśniewski, Katarzyna Roszek

**Affiliations:** 1 Physicochemistry of Carbon Materials Research Group, Faculty of Chemistry, Nicolaus Copernicus University in Toruń, Gagarina 7, 87-100 Toruń, Poland; 2 Department of Biochemistry, Faculty of Biological and Veterinary Sciences, Nicolaus Copernicus University in Toruń, Lwowska 1, 87-100 Toruń, Poland

**Keywords:** nano-TiO_2_, anatase, amorphous, photoactivity, cytotoxicity, in situ FTIR

## Abstract

Titanium dioxide is one of the best described photosensitive materials used in photocatalysis, solar cells, self-cleaning coatings, and sunscreens. The scientific and industrial attention has been focused on the highly photoactive crystalline phase of titanium dioxide (TiO_2_). It is commonly accepted that the smaller TiO_2_ particles, the higher photoactivity they present. Therefore, titanium dioxide nanoparticles are massively produced and widely used in everyday products. The amorphous phase of titanium dioxide has been treated with neglect, as the lack of its photocatalytic properties is assumed in advance. In this work, the complex experimental proof of the UV-protective properties of the nano-sized amorphous TiO_2_ phase is reported. Amorphous n-TiO_2_ is characterized by photocatalytic inactivity and, as a consequence, low cytotoxicity to fibroblast cells. When exposed to UV radiation, cells with amorphous TiO_2_ better survive under stress conditions. Thus, we postulate that amorphous n-TiO_2_ will be more beneficial and completely safe for cosmetic applications. Moreover, the results from in situ FTIR studies let us correlate the low toxicity of amorphous samples with low ability to form hydroperoxo surface species.

## 1. Introduction

Titanium dioxide (TiO_2_) seems to be a well-characterized material used in many branches of industry because of its photocatalytic properties. The majority of studies on TiO_2_ concern the crystalline phases (anatase, rutile, and brookite) that are known to be highly photoactive, stable, and noncorrosive, and are, therefore, largely used in industrial and consumer products [1]. The phenomena of titania material photoactivity is well known and proved. UV irradiation induces electron–hole pairs on the material surface, which can react with water or oxygen particles, or some other individuals, such as hydroxyl ions. Generated molecules of free radicals and other reactive oxygen species can interact, e.g., with pollutants to neutralize them. Currently, both rutile and anatase are presented in a number of publications as pure, doped, or composite materials, and every single modification leads to improving their photocatalytic activity in UV or visible light, or expanding their pollution removal efficiency with more chemical substances [2].

At the same time, information about properties of amorphous TiO_2_ is almost not reported at all. The lack of its photoactivity is attributed to the presence of numerous defects in the amorphous phase, which can lead to rapid recombination of photogenerated electrons and holes before they can be involved in reactions [3]. Data confirming photocatalytic activity of this material are rare, but studies are carried out to induce this activity or prove that this process can occur spontaneously [3]. The doping with some elements or creating a composite material can increase the efficiency of active center formation and slow down the recombination process, but pure amorphous titanium dioxide has no reasonable capabilities to be photocatalytically active. Nevertheless, the inactivity of amorphous titanium dioxide seems to still be a controversial issue. 

Why are the reports of photocatalytic activity or inactivity of amorphous TiO_2_ under UV radiation not conclusive? A lot depends on the conditions of the photocatalytic experiment. The crystalline phase is quite easily formed during heating at the temperature starting from as low as 100 °C [4]. Simultaneously, under intensive UV irradiation, a surface transformation into rutile takes place. Therefore, even locally appearing extreme conditions, inter alia, temperature increase, can induce phase transition and changes in the amorphous TiO_2_ structure and its photocatalytic properties. Moreover, when the size of TiO_2_ is reduced to nanoscale, the physicochemical properties and bioactivity of nano-sized TiO_2_, crystalline or not, significantly differ from the properties of its bulk analogue [5,6]. The increased photocatalytic activity of titanium dioxide nanoparticles (TiO_2_ NPs) is their most important advantage. Therefore, these NPs are massively produced and widely used in everyday life. On the other hand, they have posed potential risk to human health due to their increased photoactivity combined with a wide range of applications [7]. 

Currently, there are numerous in vitro and in vivo studies investigating the toxic effects of TiO_2_ NPs (summarized in [8]). Much experimental evidence suggests that exposure to titanium dioxide could be harmful and cause negative health effects. In vitro studies showed that TiO_2_ NPs are cyto- and genotoxic [9], can lead to fast necrosis [10], inflammation [11], induced reactive oxygen species (ROS) production [12], and altered antioxidant capacity [13], leading to cell death. However, the results are often inconsistent, probably as a consequence of the large variety of nano-sized TiO_2_ forms, obtained under distinct conditions, in different crystalline phases, and exhibiting altered physicochemical characteristics [14,15]. Among others, the ROS-generating activity of TiO_2_ was reported to be size- and crystal-phase-dependent [16].

In recent decades, there are more and more cases of skin cancer diagnosed every year. There is no doubt that UV radiation contributes significantly to carcinogenesis. In the sun-protective creams or emulsions, there are two types of anti-UV filters: chemical and physical. The chemical agents absorbing UV radiation often irritate the skin; they can also cause allergic reactions. According to the EU regulations, TiO_2_ is considered as a low toxic agent and accepted to be used in cosmetics as colorant and a physical UV filter at concentrations up to 25% [8,17]. However, the TiO_2_ introduced into sunscreen formulations is usually crystalline, and more and more often nanosized, since reducing the particle size improves spreadability and provides transparency to titanium dioxide [8,15]. As mentioned above, TiO_2_ forms differing in size and crystallinity exhibit altered physicochemical and toxicological properties. One can expect, that nano-TiO_2_ will produce ROS that are inducers of cell membrane, protein, and DNA damage, accelerating skin ageing. Many literature reports have been focused on decreasing the photocatalytic properties through organic “coating” to make TiO_2_ safer and biocompatible [18]. Instead of this, the amorphous titanium dioxide can become a natural replacement of crystalline forms, with the same protective properties but without phototoxicity.

The aim of the presented work was to prove and experimentally explain on a molecular level the photocatalytic inactivity and, consequently, low cytotoxicity of amorphous n-TiO_2_ phase. The set of comprehensive experiments evidences the satisfactory UV-protective properties with the lack of photocatalytic activity and, consequently, the lowered cytotoxicity of amorphous n-TiO_2_ towards model fibroblast cells. Thus, we postulate that amorphous n-TiO_2_ is more beneficial and safer than crystalline phases for photoprotective applications.

## 2. Results and Discussions

**Figure 1:**
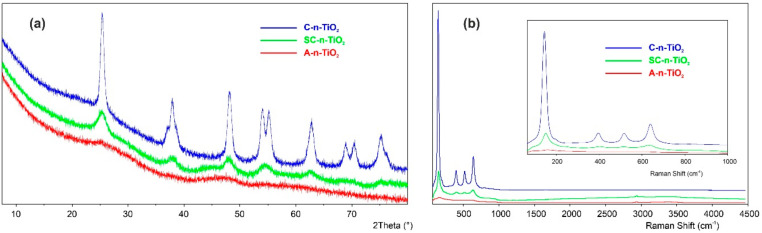
XRD (**a**) and Raman (**b**) characteristics of synthesized n-TiO_2_ materials.

The capability of synthesis of different TiO_2_ forms has been proved and summarized in Figure 1. From Raman as well as XRD investigations (Figure 1a,b), it is obvious that crystalline phase starts to form during drying already at 120 °C, which is visible as small and broad signals. Drying the samples at room temperature causes the crystalline phase to not appear. Oppositely, during annealing, in the samples at higher temperatures, i.e., 550 °C, apparent signals attributed to TiO_2_ in anatase form are observed.

**Figure 2:**
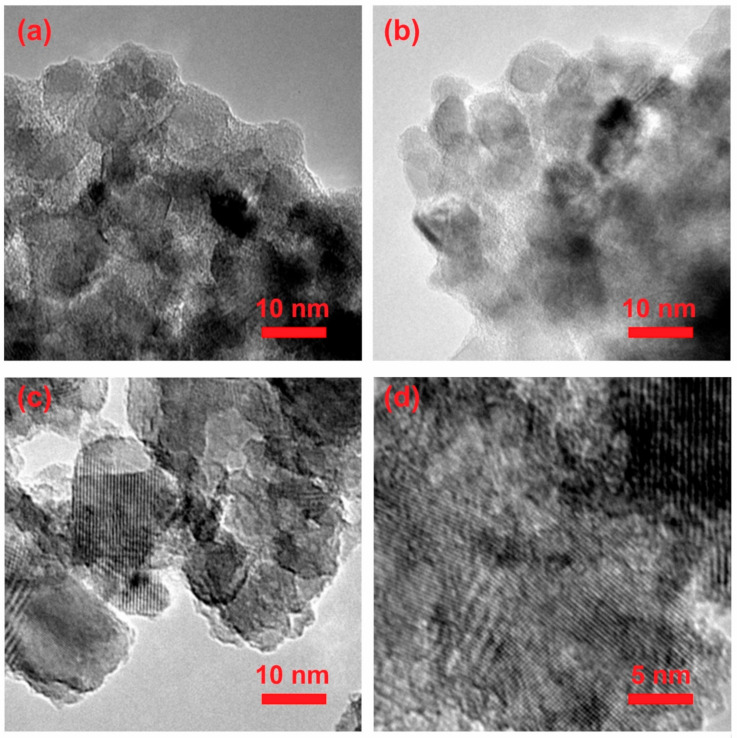
HRTEM pictures of synthesized n-TiO_2_: (**a**) amorphous, (**b**) semicrystalline, (**c**,**d**) crystalline.

HRTEM investigations confirm the above observations (Figure 2). Material dried at 30 °C (Figure 2a) is completely amorphous, without any characteristic crystal structures. These appear already after annealing at 550 °C (Figure 2d). 

Interestingly, the low temperature N_2_ adsorption isotherm analysis reveals the changes in the specific surface area and pore size distribution. S_BET_ being as low as 8 m^2^/g after drying at 30 °C increases up to ca. 250 m^2^/g due to drying at 120 °C. Annealing at 550 °C causes a decrease in pore sizes and S_BET_ (Table 1).

**Figure 3:**
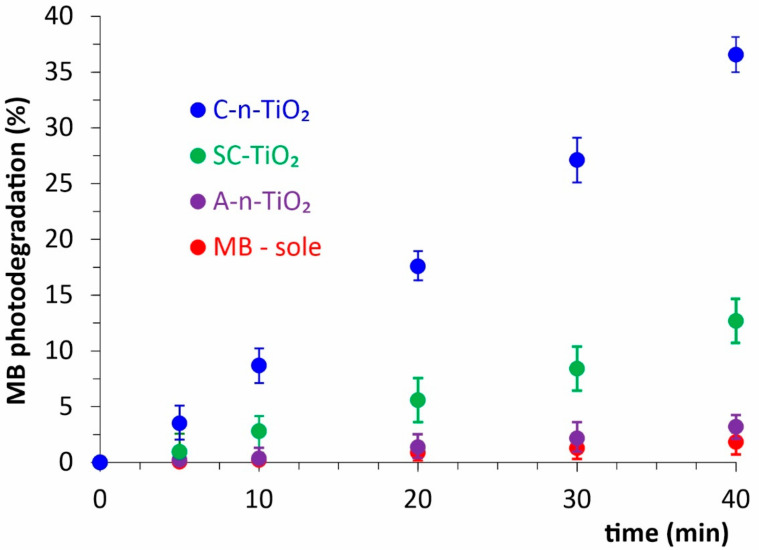
The photocatalytic properties of synthesized n-TiO_2_ during Methylene Blue (MB) degradation.

The photocatalytic properties of synthesized samples with different crystallinity are compared in Figure 3. The degree of MB self-photodegradation, i.e., without the addition of any catalysts, was as small as 2% after 40 min irradiation. At the same time, the addition of amorphous material (A-n-TiO_2_) increased the degradation only up to 3.5%. A significant increase in photocatalytic properties was observed for SC-n-TiO_2_, as well as for C-n-TiO_2_ samples. For the latter, MB was degraded 36% after 40 min irradiation.

From the above results, it is obvious that, in comparison to C-n-TiO_2_, the photocatalytic properties of the A-n-TiO_2_ sample are negligibly small. Thus, the material should exhibit rather photoprotective abilities instead of photocatalytic properties.

**Figure 4:**
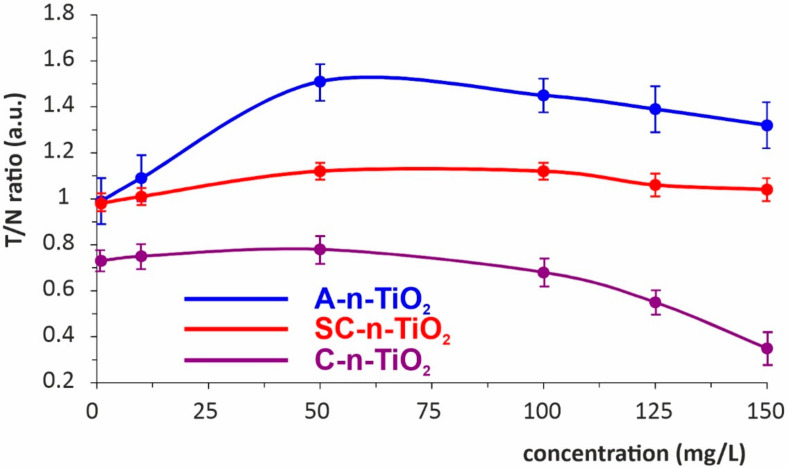
The concentration-dependent photoprotective effect of n-TiO_2_ on fibroblast cells irradiated with UVB. The cell viability was assayed with MTT test and expressed as the ratio of viable cells treated with n-TiO_2_ to the not treated control (T/N), both of them irradiated with UV.

In order to confirm the photoprotective abilities of tested materials, the in vitro experiment with fibroblasts (3T3 cell line) was performed. The cells were exposed to UVB irradiation in the presence of n-TiO_2_ specimens or without TiO_2_ treatment. The results concerning the cell viability are expressed as the ratio of viable cells treated with n-TiO_2_ suspension to the not treated control (T/N), both of them irradiated with UV. The results presented in Figure 4 estimate the photoprotective activity of amorphous comparing to crystalline phases of n-TiO_2_. The T/N ratio for a crystalline TiO_2_ sample was stable up to 50 µg/mL at the level of 0.8. The further increase in TiO_2_ concentration caused a significant decrease in T/N ratio, indicating the higher cytotoxicity of radiation-activated material. Oppositely, for the SC-n-TiO_2_ sample, the high T/N ratio slightly over unity was observed, independently of the concentration, in the whole tested range. The most beneficial photoprotective properties were observed for A-n-TiO_2_ material. The T/N ratio in this case has been maintained between 1.5 and 1.32 for cells cultured in the presence of 50–150 µg/mL TiO_2_, indicating the better survival of fibroblasts irradiated with UV in the presence of at least 50 µg/mL A-n-TiO_2_.

The above results confirm the previously reported and generally accepted low toxicity of crystalline TiO_2_ in a low concentration range [19]; nevertheless, they proved its toxic effect at higher concentrations specifically after UV-mediated activation.

To explain the observed phenomena, one needs to look closer to the surface structures present on the catalyst molecules. Here, we harnessed the in situ FTIR spectroscopy as the reasonable solution.

**Figure 5:**
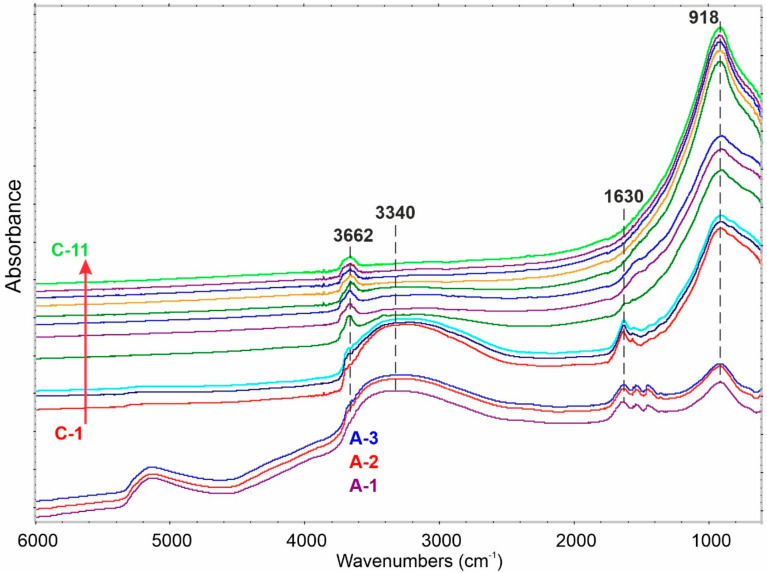
The FTIR spectra of A- and C-n-TiO_2_. A-1: A-n-TiO_2_ in air at 25 °C, A-2: A-n-TiO_2_ in He at 50 °C for 3 h, A-3: A-n-TiO_2_ in He at 50 °C for 24 h; C-1: C-n-TiO_2_ in air at 25 °C, C-2: C-n-TiO_2_ in He at 50 °C for 3 h, C-3: C-n-TiO_2_ in He at 50 °C for 24 h. C-4 to C-11 were exposed to He at temperatures ranging from 200 to 550 °C by using 50 °C steps.

The FTIR spectrum of the anatase form of n-TiO_2_ (Figure 5, C1–C11) after exposition to air (C1) is specific by three groups of signals: (i) wide and mutually overlapped in OH stretching region; (ii) δ(OH) at 1630 cm^−1^; and (iii) clear signal at 918 cm^−1^. While signals (i) and (ii) are directly connected with H_2_O existence on the TiO_2_ surface, the latter should be linked to surface oxygen bridges (Ti–O–Ti). Similar groups of IR signals were found for A-n-TiO_2_ material. Unremoved substrate/solutes are visible as small signals at 1515 and 1444 cm^−1^. Additionally, water forms a liquid film (represented by the band at 5140 cm^−1^) around the amorphous A-n-TiO_2_, which suppresses via shielding the intensity of the 918 cm^−1^ band (Figure 5, A-1).

The attempt to remove water via purging the samples in He stream at 50 °C, even for 24 h, is not efficient (Figure 5, A-3 and C-3). It proves strong interactions between TiO_2_ surface and water molecules. It is worth noting that the film is irremovable under these conditions.

The removal of H_2_O from anatase n-TiO_2_ starts to be effective already at temperatures above 200 °C (Figure 5, C-4), revealing the existence of free –OH and H-bonded –OH, respectively, at 3670 (sharp) and at ca. 3200 cm^−1^ (low and wide). Total water exclusion is possible already over 550 °C.

While performing the UV irradiation in H_2_O with the use of TiO_2_ the hydroxyl radicals (·OH) are expected to be formed. Their origin requires dissociative H_2_O adsorption and forming peroxo (–O–O–) and hydroperoxo (–OOH) functionalities. 

Generally, the assignment of O–O stretching vibrational frequencies observed for peroxo and hydroperoxo species is ambiguous and depends on the nature of the surface [20]. In general, the O–O stretching IR signals for peroxo species are broad and observed in a wide frequency range. The O–O stretches observed at 940–820 cm^−1^ and 800–740 cm^−1^ on H_2_O_2_-treated TiO_2_ were attributed to stretching vibrations of the O–O and O–OH bonds, respectively [21]. Munuera et al. [22] assigned the peaks in the range 800–932 cm^−1^ to the O–O stretching mode of peroxo species. Moreover, Nakamura et al. [23] reported the peak at 933 cm^−1^ for surface peroxo and 828 cm^−1^ for hydroperoxo species.

**Figure 6:**
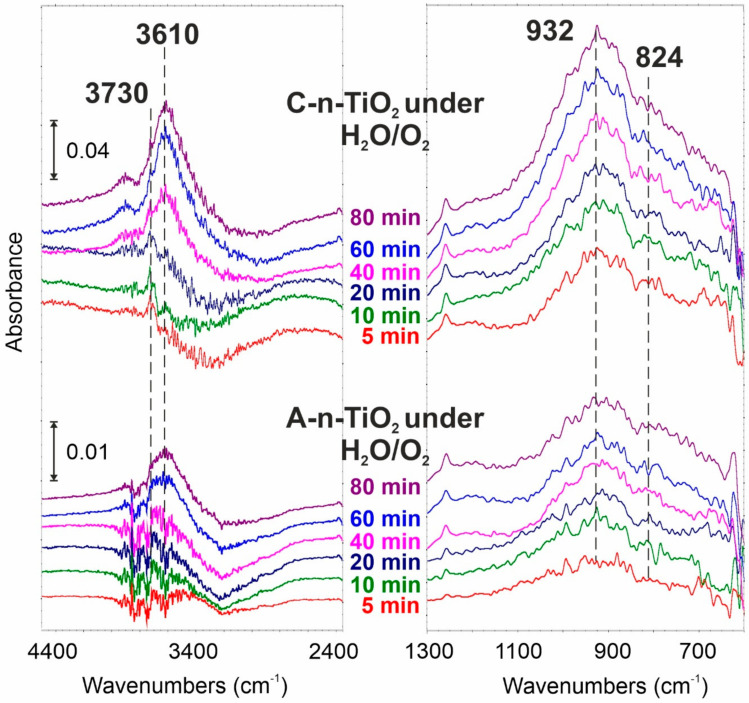
Spectral changes registered after A-n-TiO_2_ and C-n-TiO_2_ exposition to H_2_O/O_2_ atmosphere at 25 °C.

Surface hydroperoxo (–O–O–H) has been identified as an active species when n-TiO_2_ was exposed to H_2_O/O_2_ gas mixture under UVB irradiation. It is observed as a positive band of free OH stretch at 3620 cm^−1^, and negative at 3200 cm^−1^. Additionally, the rise of IR peaks at 930 and 830 cm^−1^ are present (Figure 6). Thus, clear differences between amorphous and anatase forms are observed. The spectral changes in amorphous n-TiO_2_ are much smaller than in anatase n-TiO_2_, resulting from different water affinity and, thus, different H_2_O splitting possibilities under UV irradiation. 

These physicochemical properties can underlie the described results on activity/bioactivity and explain the smaller photocatalytic properties of amorphous n-TiO_2_ material.

Despite some controversies concerning n-TiO_2_ cyto/genotoxicity, it is approved by the FDA and European Commission to be used in the food and cosmetic industries [1,2,19]. Industrial release of n-TiO_2_ at high concentrations is a major concern in aquatic environments [24]. Even in the recent review article on n-TiO_2_ ecotoxicity, the authors focused on the crystalline forms, while the amorphous form is still underestimated. Knowing this, we would like to draw the attention to a safer alternative solution, which is amorphous n-TiO_2_.

## 3. Materials and Methods

### 3.1. Synthesis of Nanostructural TiO_2_

All used reagents were purchased from Sigma-Aldrich, Darmstadt, Germany. Nano-TiO_2_ (n-TiO_2_) has been synthesized with the sol–gel method from titanium isopropoxide as a precursor via an acid-catalyzed hydrolysis step, followed by condensation (as described in [25]). Briefly, a mixture of 0.1 mL conc. HCl and 0.3 mL of glacial acetic acid in 10 mL of isopropanol was prepared. To this solution, 1 mL of titanium isopropoxide was added under continuous stirring. The hydrolysis was performed by careful addition, drop by drop, of 8 mL of H_2_O. 

Drying the obtained samples at 30 °C under vacuum for 24 h lead to amorphous material formation (A-n-TiO_2_). To obtain semicrystalline and crystalline material, i.e., anatase, samples were calcined at 120 °C for 24 h (SC-n-TiO_2_) and 550 °C for 5 h (C-n-TiO_2_) under air, respectively.

### 3.2. The n-TiO_2_ Characterization

Produced as described above, n-TiO_2_ samples were fully characterized by different methods:

- High-resolution transmission electron microscopy (HRTEM): the images were taken using a transmission electron microscope F20X-TWIN (FEI-Tecnai) operated at 200 kV. The drop of sample solution was placed on a Cu grid coated with an ultrathin amorphous carbon film, and then dried under ambient condition.
- UV–vis absorption: the assay was carried out using Jasco 660 UV–vis spectrophotometer. The apparatus was also used for methylene blue (MB) photo-degradation study (c_o_ = 20 µmol/L, irradiation: 360 nm, 0.6 W·cm^−2^).
- The Fourier transform infrared (FTIR) measurements: spectra were accomplished by Bruker Vertex 70 infrared spectrophotometer using drift mode techniques in the frequency range 400–6000 cm^−1^.
- The Raman measurements: the nonpolarized spectra of carbon structures were investigated in the spectral range of 60−4500 cm^−1^. Raman spectra were recorded in the backscattering geometry using SENTERRA micro-Raman system. As an excitation light, we used the green laser operating at 532 nm. The laser beam was tightly focused on the sample surface through a 30× microscope objective. To prevent any damage of the sample, an excitation power was fixed at 20 mW. The resolution was 4 cm^−1^ and CCD temperature of 223 K, laser spot of about 10 μm, and total integration time of 100 s (50 × 2 s) were used. The position of the microscope objective with respect to the sample was piezoelectrically controlled (XY position).
- The bulk powder samples were characterized with XRD using a Philips XPERT Pro diffractometer with CuKα1 radiation.
- Nitrogen adsorption–desorption isotherms were measured using an ASAP 2010 volumetric adsorption analyzer from Micromeritics (Norcross, GA, USA) at liquid nitrogen temperature (77 K) in the relative pressure range from about 10^–6^ up to 0.999. Before the measurements, the samples were outgassed for at least 2 h at a temperature of 50 or 393 K. Low desorption temperature was used for amorphous n-TiO_2_ to avoid the crystalline phase formation.

Caring for the identical samples’ preparation circumstances, i.e., the same support, surface concentration, drop volume, and drying conditions, as well as the measurement conditions, we were able to compare RS and XRD signal intensities.

### 3.3. In Vitro Cell Culture

The 3T3 fibroblast cell line was purchased from ATCC collection. Cells were grown in DMEM-LG medium containing 2 mM glutamine, 10% fetal bovine serum (FBS), and 1% penicillin/streptomycin at 37 °C in a CO_2_ incubator with 5% CO_2_. A volume of 25 μL containing approximately 1 × 10^5^ cells was seeded to each well of a 6-well plate 24 h before the experiment was started. 

### 3.4. Cytotoxicity Experiments

n-TiO_2_ at different concentrations in the range of 1–150 µg/mL was added to the growing fibroblasts just before their exposure to UV radiation (311 nm, 3–5 min, 0.1 W∙cm^−2^), while control cells were left without n-TiO_2_, similarly to our previous study [26]. After the UV treatment, the cells were incubated for the next 24 h. Subsequently, the MTT test, based on the ability to reduce 3-(4,5-dimethylthiazol-2-yl)-2,5-diphenyltetrazolium bromide (MTT) by mitochondrial dehydrogenases was performed in triplicate for assessing cell metabolic activity and viability. The plates were then read spectrophotometrically at a wavelength of 570 nm to measure the amount of reduced formazan.

### 3.5. Statistical Analysis

All experiments were performed in a 3-times triplicate formula, i.e., three biological repetitions, each in three sample wells. The mean values of T and N, as well as their standard deviations ΔT and ΔN, were calculated for the obtained results. The standard deviation of the ratio T/N was calculated according to the following equation:

$$\Delta \left(\frac{T}{N}\right)=\frac{T}{N}\;\sqrt{{\left(\frac{\Delta T}{T}\right)}^{2}+{\left(\frac{\Delta N}{N}\right)}^{2}}$$

## 4. Conclusions

As highlighted in the introduction, we aimed to explain on a molecular level the photocatalytic inactivity and consequently low cytotoxicity of A-n-TiO_2_. Based on the obtained results, we were able to correlate the low toxicity of amorphous phase with the low ability to form hydroperoxo surface species. 

In conclusion, the amorphous nano-TiO_2_ particles presented negligibly small photocatalytic properties and, as a consequence, low cytotoxicity to fibroblast cells. When exposed to UV radiation, cells cultured with A-n-TiO_2_ better survive under stress conditions, confirming the photoprotective ability of A-n-TiO_2_. Thus, we postulate that amorphous nano-TiO_2_ will be a safe photoprotective agent for cosmetic applications. Since the release of high concentrations of n-TiO_2_ into the aquatic environment is one of the major concerns, A-n-TiO_2_ deserves more scientific attention and further research as a safer alternative. We believe that our results shed light on the perspective of using amorphous TiO_2_ in such applications.

## Figures and Tables

**Table 1:** The surface area of obtained n-TiO_2_ samples.

Sample	Surface Area [m^2^/g]
A-n-TiO_2_	8
SC-n-TiO_2_	248
C-n-TiO_2_	109

## Data Availability

Not applicable.
